# Rotator Cuff Surgery and Artificial Intelligence: A Comparative Analysis of Quality and Readability Between ChatGPT and Gemini

**DOI:** 10.1055/s-0046-1820460

**Published:** 2026-06-16

**Authors:** Vinicius Borges Alencar, Matheus Mariano Teles, Diogo Fonseca da Cunha, Pedro Henrique Nunes Piropo, Paulo Sergio Milan Robazzi, Thiago Batista Faleiro

**Affiliations:** 1 Faculdade de Medicina da Bahia, Universidade Federal da Bahia, Salvador, BA, Brazil; 2 Universidade Federal do Triângulo Mineiro, Uberaba, MG, Brazil; 3 Hospital São Rafael, Salvador, BA, Brazil; 4 Hospital Santo Antônio, Obras Sociais Irmã Dulce (OSID), Salvador, BA, Brazil

**Keywords:** artificial intelligence, ChatGPT, health education, information technology, language models, rotator cuff, ChatGPT, educação em saúde, inteligência artificial, manguito rotador, modelos de linguagem, tecnologia da informação

## Abstract

**Objective:**

Artificial intelligence (AI) tools based on natural language, such as ChatGPT 4.1 mini (OpenAI Group PBC) and Gemini 2.5 Flash (Alphabet Inc.), are used by patients as sources of medical information. The current study aimed to evaluate and compare the quality and readability of responses provided by these AIs, in Brazilian Portuguese, regarding rotator cuff surgery.

**Methods:**

The present cross-sectional, descriptive, and comparative study followed qualitative and quantitative approaches. A total of 24 frequently-asked patient questions were used, classified according to Rothwell. Each question was entered individually into both platforms, and only the first response was considered. The quality assessment used the DISCERN instrument, developed by the University of Oxford and the British Library, and the Journal of the American Medical Association (JAMA) benchmark criteria. Readability was estimated using Análise de Legibilidade Textual (ALT, “Text Readibility Anallysis”, in Portuguese) software, validated for Brazilian Portuguese. The statistical analyses included the Wilcoxon and Friedman tests, repeated-measures analysis of variance (ANOVA), and the Conover post-hoc test with Bonferroni correction.

**Results:**

ChatGPT achieved a mean DISCERN score of 58.7 ± 4.0, and Gemini, 56.3 ± 3.5, with no significant difference ( *p* = 0.174), but with a maximum effect size (rank-biserial correlation [rrb] = 1.0). Both models showed a mean readability corresponding to 13.3 years of schooling ( *p* = 1.000). No response met the JAMA benchmark criteria. Value-based questions achieved the highest quality scores, whereas policy-related questions were the most complex in terms of readability. The correlation between quality and readability was moderate (ρ = 0.73; *p* = 0.099).

**Conclusion:**

ChatGPT 4.1 mini and Gemini 2.5 Flash do not yet provide adequate medical information in Brazilian Portuguese regarding editorial reliability, quality, and textual accessibility for the general public.

## Resumo

**Objetivo:**

Ferramentas de inteligência artificial (IA) baseadas em linguagem natural, como ChatGPT-4.1 mini (OpenAI Group PBC) e Gemini 2.5 Flash (Alphabet Inc.), são utilizadas por pacientes como fonte de informação médica. Este estudo avaliou e comparou a qualidade e a legibilidade das respostas fornecidas por essas IAs, em português brasileiro, sobre cirurgia do manguito rotador.

**Métodos:**

Estudo transversal, descritivo e comparativo, com abordagem qualiquantitativa. Foram utilizadas 24 perguntas frequentes de pacientes, classificadas segundo Rothwell. Cada pergunta foi inserida individualmente nas plataformas dos dois modelos, sendo considerada apenas a primeira resposta. A qualidade foi avaliada por meio do instrumento DISCERN, desenvolvido pela University of Oxford e pela British Library, e dos critérios editoriais da Journal of the American Medical Association (JAMA). A legibilidade foi estimada com o programa Análise de Legibilidade Textual (ALT), validado para o português brasileiro. As análises estatísticas incluíram os testes de Wilcoxon, Friedman, análise de variância ([ *analysis of variance* , ANOVA, em inglês] para medidas repetidas) e *post hoc* de Conover com correção de Bonferroni.

**Resultados:**

O ChatGPT obteve escore médio DISCERN de 58,7 ± 4,0, e o Gemini, 56,3 ± 3,5, sem diferença significativa ( *p* = 0,174), mas com efeito máximo ( *rank-biserial correlation* [rrb, em inglês] = 1,0). Ambos os modelos apresentaram legibilidade média correspondente a 13,3 anos de escolaridade ( *p* = 1,000). Nenhuma resposta atendeu aos critérios editoriais da JAMA. Perguntas relacionadas a valores obtiveram os maiores escores de qualidade, ao passo que as perguntas sobre política foram as mais complexas em termos de leitura. A correlação entre qualidade e legibilidade foi moderada (ρ = 0,73; *p* = 0,099).

**Conclusão:**

ChatGPT–4.1 mini e Gemini 2.5 Flash ainda não oferecem informação médica, em português brasileiro, adequada quanto à confiabilidade editorial, qualidade e acessibilidade textual para o público leigo.

## Introduction

Natural-language models based on artificial intelligence (AI), such as ChatGPT (OpenAI Group PBC) and Gemini (Alphabet Inc.), have been widely used by patients for online searches on medical topics, including orthopedic conditions such as rotator cuff injuries. This immediate accessibility transforms the traditional process of health education but raises concerns regarding the quality, editorial reliability, and accessibility of the content generated by these tools. 1 2

Recent studies 3 4 5 demonstrate that, although texts generated by large language models (LLMs) are well structured and semantically coherent, they often lack fundamental elements of traceability, such as identifiable authorship, date of last update, and valid bibliographic references, which compromises their editorial reliability. In addition, the language used tends to be excessively complex, hindering comprehension among patients with lower levels of schooling, which contradicts international readability guidelines for health education materials. 6 7

A recurring methodological limitation in the literature is the use of readability tools developed for the English language, which limits their applicability in Portuguese-speaking contexts. To address this limitation, Análise de Legibilidade Textual (ALT, “Text Readability Analysis”, in Portuguese) software was developed and validated for Brazilian Portuguese, and it enables a more accurate estimation of the minimum level of schooling required for text comprehension. 8

Although the use of generative AI as a source of medical information has progressively expanded in Brazil, there are still no systematic studies evaluating the quality and readability of responses in Brazilian Portuguese provided by free versions of these tools. In light of this gap, the present study aims to compare the informational quality and readability of responses generated by the ChatGPT 4.1 mini and Gemini 2.5 Flash models, in Brazilian Portuguese, regarding rotator cuff repair surgery, a frequent topic in the orthopedic practice and of high interest to the general public.

## Methods

### Study design

The current cross-sectional, descriptive, and comparative study followed quantitative and qualitative approaches to evaluate and compare the quality and readability of information provided by two free AI models based on natural language: ChatGPT 4.1 mini and Gemini 2.5 Flash. Since the study did not involve human or animal subjects, it did not require approval from the Research Ethics Committee.

### Selection of AI models

The free versions of these tools were accessed directly at the official platforms: https://chat.openai.com and https://gemini.google.com . No customized settings, contextual instructions, or plug-ins were used. The tests were performed between July 1st and July 10th, 2025.

### Selection and classification of questions

We selected 24 questions commonly asked by patients in an outpatient setting regarding rotator cuff surgical repair, based on the study by Warren et al *.* 3 ( Table 1 ). The questions were translated to Brazilian Portuguese and reviewed by two specialists with experience in orthopedics and health communication to ensure semantic and cultural equivalence. Next, they were classified according to the system proposed by Rothwell 9 into three argumentative categories: fact questions (objective questions aimed at determining whether something is true and to what extent, and which can be answered based on objective evidence), policy questions (related to therapeutic decisions), and value questions (related to subjective perceptions or preferences), with eight questions in each category.

**Table 1:** Complete list of questions classified according to Rothwell, 9 adapted from Warren et al. 3

Fact	Policy	Value
**1. Can an X-ray show a rotator cuff injury?**	1. What happens if a rotator cuff injury is not treated?	1. Is arthroscopic shoulder surgery worth it?
**2. How do I go to the bathroom after shoulder surgery?**	2. Is it possible to wait too long to undergo rotator cuff surgery?	2. Why is rotator cuff surgery so painful?
**3. How can I tell if I have torn my rotator cuff?**	3. Can a rotator cuff injury heal on its own?	3. How long does a rotator cuff repair last?
**4. How much does rotator cuff surgery cost?**	4. How can I speed up recovery after rotator cuff repair?	4. Why does rotator cuff pain worsen at night?
**5. Can a bra be worn after shoulder surgery?**	5. Does rotator cuff surgery prevent the development of osteoarthritis?	5. Will rotator cuff surgery restore my shoulder to its previous condition?
**6. How long after shoulder surgery can I drive?**	6. What should I do to return to sports after rotator cuff repair?	6. Should I still undergo rotator cuff surgery if I cannot afford physical therapy?
**7. How long is it necessary to sleep in a chair after shoulder surgery?**	7. What happens if I do not undergo physical therapy after rotator cuff surgery?	7. Can I undergo rotator cuff surgery even if I do not have someone to help me at home?
**8. What is the average recovery time after rotator cuff surgery?**	8. How long can I wait before undergoing rotator cuff surgery?	8. Is rotator cuff surgery a major procedure?

### Generation and organization of responses

Each of the 24 questions in Brazilian Portuguese was individually entered into the web interfaces of both AI models. The analysis considered only the first response generated by each model. The responses were transcribed in full, without editing, reformulation, or exclusion of content, and stored in a structured digital form. For each entry, the following were recorded: question identification, classification according to Rothwell, 9 AI model used, full response text, quality assessment scores, and readability index.

### Assessment of response quality

Two independent reviewers experienced in critical evaluations of scientific literature assessed the quality of the responses. This analysis used two validated instruments: the DISCERN questionnaire 10 and the *Journal of the American Medical Association* (JAMA) benchmark criteria. 11 12

The DISCERN questionnaire was developed in 1999 by the British Library and the National Health Service Research and Development Programme to assess the quality of written health information available to the public. 10 It is a validated tool widely used in the scientific literature to evaluate the reliability and usefulness of health education materials. It consists of 16 items divided into 3 sections: 1) reliability of the information (8 items); 2) quality of information related to treatment options (7 items); and 3) an overall quality rating of the text (1 item). Each item is scored on a Likert scale from 1 (“does not meet criteria”) to 5 (“fully meets criteria”), resulting in a total score ranging from 16 to 80. Scores above 70 are typically categorized as “excellent” quality, while scores above 50 are considered “good” quality. The assessments were performed independently by two reviewers blinded to the identity of the AI model. Disagreements were resolved by consensus after joint reassessment

The JAMA benchmark criteria include four essential domains for the reliability of health information: authorship, attribution of sources, disclosure of conflicts of interest, and indication of the date of last update. Each criterion was assessed in a binary manner (present = 1 point; absent = 0 points), resulting in a total score ranging from 0 to 4. 11

### Assessment of response readability

The ALT software, a tool developed in and validated for Brazilian Portuguese, assessed text readability. 8 The software applies adapted classical formulas, such as Flesch Reading Ease, Gunning Fog, Automated Readability Index (ARI), and Coleman–Liau, estimating the minimum level of schooling required for text comprehension. 8

### Statistical analysis

Statistical analyses were performed using Jeffreys's Amazing Statistics Program (JASP, free and open source), version 0.19.3.0. Continuous variables were described as mean and standard deviation values. The DISCERN scores and readability indices of the models were compared using the Wilcoxon test for paired samples, with effect size estimated by the rank-biserial correlation (rrb; 95%CI). Readability across question types was compared using repeated-measures analysis of variance (ANOVA). Differences between question categories for DISCERN scores were analyzed using the Friedman test, followed by Conover post-hoc tests with Bonferroni and Holm corrections. Inter-rater reliability was assessed using the intraclass correlation coefficient (ICC; mixed-effects model, type 1,1). The significance level was set at *p* < 0.05.

## Results

### Reliability of assessment and editorial reliability

Inter-rater reliability in the application of the DISCERN instrument was considered excellent, with an intraclass correlation coefficient (ICC1,1) of 0.975 (95%CI: 0.859–0.996), demonstrating high consistency between the two independent reviewers.

Regarding editorial reliability, all 48 responses analyzed received a JAMA score of zero, indicating the absence of identifiable authorship, bibliographic references, disclosure of conflicts of interest, and date of last update.

### Overall comparison between models

ChatGPT 4.1 mini achieved a mean DISCERN score of 58.7 ± 4.0, and Gemini 2.5 Flash, 56.3 ± 3.5. Although the difference did not reach statistical significance (W = 6.0; *p* = 0.174), the effect size was maximal (rank-biserial correlation [rrb] = 1.0; 95%CI: 0.554–1.000), indicating a consistent trend toward better performance by ChatGPT ( Table 2 ).

**Table 2:** Statistical comparison between models regarding informational quality and readability

**Variable**	**Statistical test**	**W**	*p* **-value**	**rrb**	**95%CI (rrb)**	**Significance**
**DISCERN** **scores**	Wilcoxon test for paired samples	6.0	0.174	1.000	0.554–1.000	Not significant ( *p* > 0.05)
**Readability (ALT)**	Wilcoxon test for paired samples	3.0	1.000	0.000	-0.840 to 0.840	Not significant ( *p* > 0.05)

Abbreviations: ALT, Análise de Legibilidade Textual (“Textual Readability Analysis”); rrb, rank-biserial correlation.

Regarding readability, both models required an average of 13.3 years of schooling to understand the generated texts. There was no significant difference between the models in this aspect (W = 3.0; *p* = 1.000), indicating a null bb (rrb = 0.0; 95%CI: -0.840 to 0.840).

**Fig. 1:**
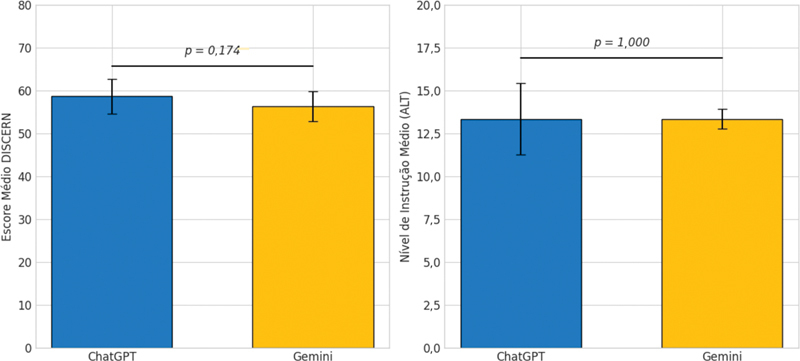
Comparison between ChatGPT 4.1 mini and Gemini 2.5 Flash. ( **A** ) Mean informational quality scores, assessed using the DISCERN instrument, for each model. ( **B** ) Mean schooling level required for the comprehension of the responses, based on the readability index of the software Análise de Legibilidade Textual (ALT, “Text Readability Analysis”, in Portuguese). No significant differences were observed between the models for either parameter ( *p* > 0.05).

Fig. 1 presents the data comparatively. Panel A displays the average DISCERN scores for each model. Panel B shows the average schooling level required based on the ALT index. In both analyses, differences between models did not reach statistical significance.

### Stratified analysis by question category

Statistical analysis categorized by the Rothwell 9 classification showed no significant differences in DISCERN scores between the groups (Friedman test, Chi-squared [χ ^2^ ] = 3.71; *p* = 0.156). Neither did the comparison of readability levels, measured using the ALT software, reveal statistically significant differences among categories (ANOVA, F = 2.11; *p* = 0.321; Table 3 ).

**Table 3:** Statistical comparison between question types according to Rothwell 9 classification

Variable	Test	Value	p-value	Correction	Significance
**DISCERN** **(overall)**	Friedman	χ ^2^ = 3.71	0.156	–	Not significant ( *p* > 0.05)
**Post hoc: Fact × Policy**	Conover	T = 3.54	0.038	Bonferroni = 0.115	Not significant after adjustment
**Post hoc: Fact × Value**	Conover	T = 4.95	0.038	Bonferroni = 0.115	Not significant after adjustment
**Post hoc: Policy × Value**	Conover	T = 1.41	0.293	Bonferroni = 0.879	Not significant
**Readability (ALT)**	ANOVA	F = 2.11	0.321	–	Not significant ( *p* > 0.05)

**Abbreviations:** χ ^2^ , Chi-squared; ALT, Análise de Legibilidade Textual **(“** Textual Readability Analysis”); ANOVA, analysis of variance.

Despite the absence of statistical significance, we observed a relevant descriptive trend: value questions achieved the highest mean quality scores (ChatGPT: 61; Gemini: 60), whereas fact questions showed the lowest values (ChatGPT: 54; Gemini: 53). Regarding readability, fact questions required a lower level of schooling (∼11–12 years), while policy questions were the most complex, reaching up to 15 years of schooling for ChatGPT responses.

**Fig. 2:**
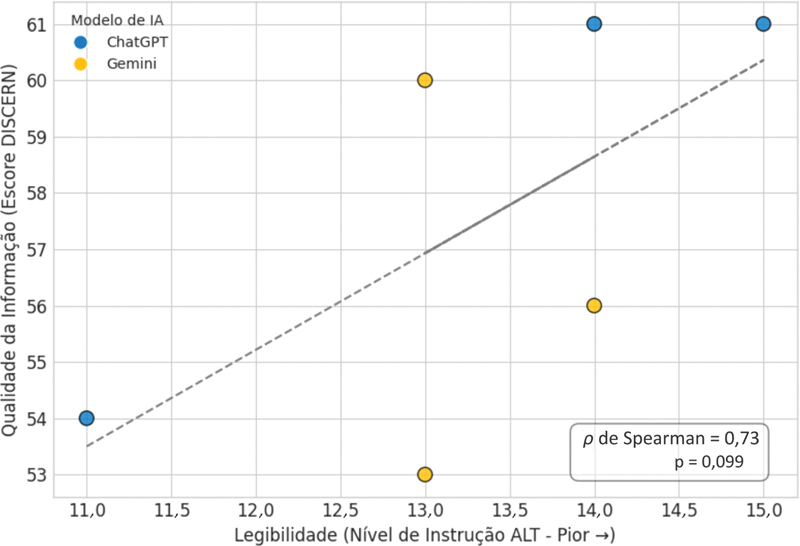
Correlation between informational quality scores (DISCERN) and readability indices (ALT) of the responses generated by ChatGPT 4.1 mini and Gemini 2.5 Flash. The analysis revealed a moderate positive correlation (ρ = 0.73), but it lacked statistical significance ( *p* = 0.099). While a relationship between quality and readability was noted, it did not achieve statistical significance due to the small sample size. **Abbreviation:** AI, artificial intelligence.

The correlation analysis between quality and readability showed a moderate positive association (ρ = 0.73; *p* = 0.099), with no statistical significance, possibly due to the limited sample size ( Fig. 2 ). The post-hoc analysis revealed a marginal difference between the “fact” and “policy” groups ( *p* = 0.038); however, this difference lost significance after the Bonferroni correction ( *p* = 0.115).

**Fig. 3:**
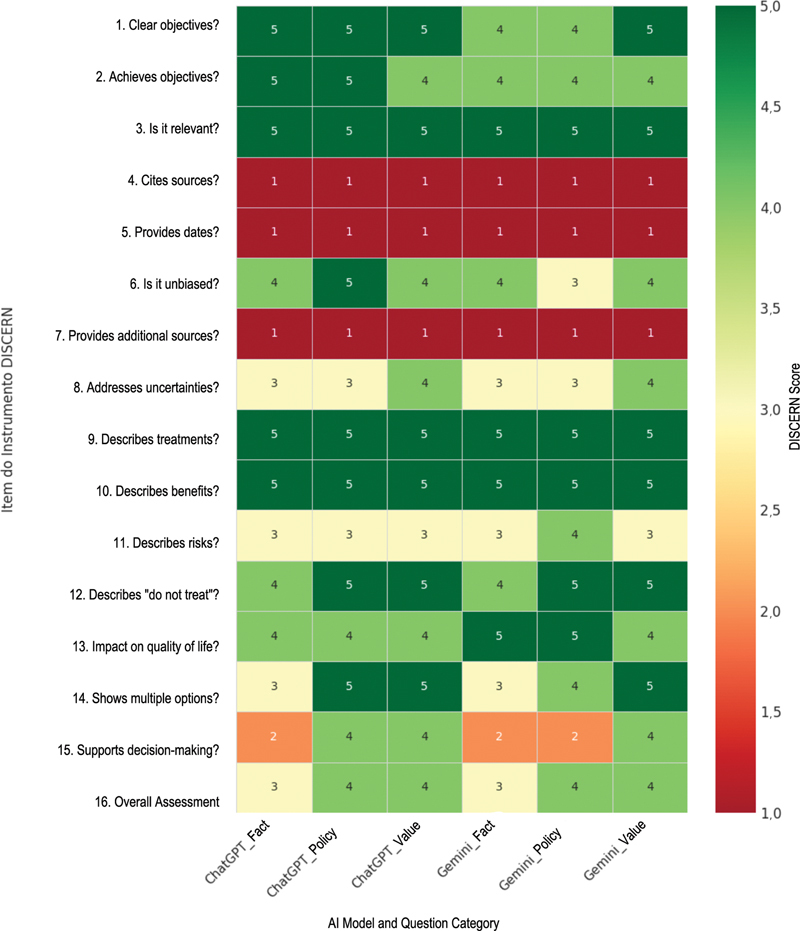
Heatmap of mean DISCERN scores by artificial intelligence (AI) model and question category (fact, policy, and value). Variations in color intensity indicate differences in quality scores, highlighting the strengths and weaknesses of each model across question types.

For a detailed visual representation of model performance by question category, Fig. 3 presents a heatmap of the mean scores obtained across the 16 items of the DISCERN instrument, highlighting the strengths and weaknesses of each AI in relation to different question types.

## Discussion

The present study identified a paradox in the use of language models in health education: the production of structured and coherent content, but with critical shortcomings in editorial reliability and accessibility. ChatGPT 4.1 mini and Gemini 2.5 Flash achieved DISCERN scores consistent with good informational quality; however, they did not meet any of the JAMA editorial criteria and required a level of schooling higher than the one recommended for the general public, which limits their practical applicability.

Although ChatGPT presented a non-significant trend toward higher scores, interpreting this difference requires caution due to the small number (n = 8) of questions per category. The methodological design, inspired by a previous study written in English 3 and defined by convenience, enabled the comparability but reduced the statistical power and increased the likelihood of type-II error, which may have masked real differences between the models.

These findings are consistent with those by Warren et al *.* 3 , who evaluated information on rotator cuff repair surgery in the English language. These authors reported DISCERN scores ranging from 51 to 55, classified as “good,” but no response met the JAMA editorial criteria (absence of authorship, date, and references). Readability, measured by the Flesch–Kincaid Grade Level (FKGL), indicated that the required schooling level falls between the 10th and 12th grades of the American high school system. This level is higher than the one recommended by the National Institutes of Health and the American Medical Association. As in the current study, the authors 3 concluded that, despite textual coherence, AI-generated content lacks traceability and linguistic accessibility, limiting its value as patient health education material.

Similarly, we observed the absence of authorship, date, and references; however, the interpretation of this finding should consider the intentional use of simplified prompts simulating patient queries. Therefore, the results reflect this specific scenario and should not be generalized to contexts employing structured medical prompts.

Gupta et al *.* 13 expanded this discussion by evaluating responses from ChatGPT 3.5 regarding distal biceps tendon surgery. The DISCERN scores ranged from 59 to 61, again classified as “good”; however, as in the present study, the JAMA editorial criteria were not met (JAMA = 0). Furthermore, the readability, assessed using the FKGL, was of approximately 15, indicating that comprehension requires university-level education. These findings reinforce the observed pattern that language models generate informationally-sound content that remains inaccessible to the general public and lacks traceability.

Giammanco et al *.* 4 analyzed six generative models—Perplexity, Copilot, ChatGPT (free and premium versions), and Gemini (free and premium versions)—using questions related to clavicle fractures and different quality metrics (DISCERN, JAMA, Global Quality Score [GQS], EQIP [Ensuring Quality Information for Patients], and readability). The authors 4 reported better performance for Perplexity and Copilot, whose responses were considered more reliable and accessible. In contrast, Gemini 1.5 Pro and the free version of ChatGPT showed the poorest results. Importantly, none of the models achieved readability below the 10th-grade level of the American high school system, corroborating our findings that textual complexity remains a barrier for patients.

The causes of this pattern appear to be related to the nature of LLMs. A possible explanation for the high textual quality is training on scientific and medical literature, which enables these models to replicate the formal structure and argumentative logic of well-developed clinical responses. 14 On the other hand, the absence of JAMA editorial criteria reflects the fact that such models are not designed to provide authorship attribution or source referencing, as their primary objective is fluent language generation rather than informational traceability. 15 Additionally, the high linguistic complexity appears to stem from the predominant stylistic register of these models, derived from their training corpus (a large collection of texts used to train the model), which is not highly responsive to simplification in the absence of explicit instructions. 16

### Clinical implications and recommendations

Language models generate coherent responses that may lead patients to overestimate their scientific validity, despite the absence of traceability and editorial criteria. This scenario highlights the critical need for healthcare professionals to thoroughly assess digital information, integrating scientific validity, clinical context, and clear, safe communication to enhance evidence-based decision-making.

### Limitations

The small number of questions limited the statistical power, restricting the generalizability of the findings. The analysis was cross-sectional, without an evaluation of the temporal consistency of the responses, and performance may vary across future model versions. In addition, there was no direct evaluation by patients, which prevents inferences regarding practical comprehension and perceived usefulness.

### Future perspectives

Future research should focus on strategies to enhance the safety and clinical applicability of AI in healthcare using currently-available models. Priorities include: 1) longitudinal studies evaluating the impact of AI-generated responses on clinical decision-making, treatment adherence, and self-management of chronic conditions; 2) validation of prompt-engineering strategies, with structured instructions that guide models to generate more accurate, comprehensible, and reliable information; and 3) analysis of approaches combining AI-generated responses with professional guidance, identifying effective ways to incorporate these tools into clinical practice.

## Conclusion

The present study showed that, in Brazilian Portuguese, the ChatGPT 4.1 mini and Gemini 2.5 Flash models generated responses on rotator cuff repair surgery with informational quality classified as “good” according to the DISCERN instrument. However, both models received a score of zero on the JAMA criteria and demonstrated readability levels above those recommended for the general public, limiting their applicability as patient health education tools.
